# The Incorporation of Host Proteins into the External HIV-1 Envelope

**DOI:** 10.3390/v11010085

**Published:** 2019-01-20

**Authors:** Jonathan Burnie, Christina Guzzo

**Affiliations:** 1 Department of Cell and Systems Biology, University of Toronto, 25 Harbord Street, Toronto, ON M5S 3G5, Canada; jonathan.burnie@mail.utoronto.ca; 2 Department of Biological Sciences, University of Toronto Scarborough, 1265 Military Trail, Toronto, ON M1C 1A4, Canada

**Keywords:** human immunodeficiency virus, host protein incorporation, integrin α4β7, major histocompatibility complex (MHC), CD54 (ICAM-1), CD4^+^ T cells, monocytes/macrophages, virion immunocapture, nanoscale flow cytometry, viral pathogenesis

## Abstract

The incorporation of biologically active host proteins into HIV-1 is a well-established phenomenon, particularly due to the budding mechanism of viral egress in which viruses acquire their external lipid membrane directly from the host cell. While this mechanism might seemingly imply that host protein incorporation is a passive uptake of all cellular antigens associated with the plasma membrane at the site of budding, this is not the case. Herein, we review the evidence indicating that host protein incorporation can be a selective and conserved process. We discuss how HIV-1 virions displaying host proteins on their surface can exhibit a myriad of altered phenotypes, with notable impacts on infectivity, homing, neutralization, and pathogenesis. This review describes the canonical and emerging methods to detect host protein incorporation, highlights the well-established host proteins that have been identified on HIV-1 virions, and reflects on the role of these incorporated proteins in viral pathogenesis and therapeutic targeting. Despite many advances in HIV treatment and prevention, there remains a global effort to develop increasingly effective anti-HIV therapies. Given the broad range of biologically active host proteins acquired on the surface of HIV-1, additional studies on the mechanisms and impacts of these incorporated host proteins may inform the development of novel treatments and vaccine designs.

## 1. Introduction

Enveloped viruses are a subset of virions that contain a lipid envelope derived from the cellular membranes of their hosts and are the etiologic agents of many of the deadliest human diseases, including Ebola, influenza, and HIV/AIDS [1,2,3]. The egress of enveloped virions from host cells is distinct from non-enveloped viruses and occurs through a budding mechanism in which virions acquire a portion of the host membrane [4,5]. During the canonical budding process, viral envelope proteins and cellular proteins that are displayed on the surface of infected cells are incorporated by nascent virions [6]. While countless studies have been performed on viral envelope proteins due to their role in viral tropism and attachment, much less literature is focused on the host cell proteins that can be hijacked by viruses and displayed on external viral membranes. Very early studies suggested that host proteins were present in the envelopes of animal viruses such as the avian myeloblastosis virus and in murine leukemia virus [7,8,9], but minimal biological significance was attributed to these findings. However, in these early studies it was noted that the host-derived ATPase proteins in the myeloblastosis virion envelope remained functionally active [7,9], demonstrating that host proteins could maintain their biological roles in viral envelopes. The incorporation of host proteins into the envelopes of animal and human retroviruses has been well established in feline leukemia virus (FeLV), simian immunodeficiency virus (SIV), and human immunodeficiency virus (HIV) [10,11,12,13,14,15].

The effects of incorporated host proteins on HIV-1 tropism and pathogenesis has been reviewed previously [16,17]. Indeed, it was suggested that host proteins incorporated into the HIV-1 envelope could alter viral pathogenesis in the early 1990s [15] and many subsequent studies have provided evidence for this. Enriched, functionally active host-derived proteins such as integrin α4β7 and ICAM-1 are key proteins of current study due to their selective incorporation into virions and their influence on viral infectivity and pathogenicity [18,19]. Antibody targeting of virus-incorporated host proteins has indicated promise in reducing or inhibiting HIV-1 and SIV infection in in vitro and in vivo experimental models [18,20,21,22,23]. Continuing to study the mechanisms by which host proteins may be selectively incorporated into viral envelopes may provide future targets for novel drug therapies and vaccine designs.

With respect to methodology, immunoelectron microscopy, which was the initial mode of detection for host proteins on the surface of virions, has now fallen out of favour as other technological advances and methodologies have emerged. Mass spectrometry (MS) is now more commonly used to identify the breadth of host proteins associated with virions [24,25,26,27,28], while immunocapture with magnetic beads is routinely employed to identify host proteins displayed on the surface of virions [18,19,29]. This review will highlight host proteins that are well-established to be incorporated into HIV-1 virions, tools used to identify host protein incorporation, and lastly, how these incorporated proteins can influence viral pathogenesis and could be targeted in novel therapeutics.

## 2. Methods to Identify Host Protein Incorporation

Despite the ubiquity of viruses, detailed knowledge of viral structures is difficult to garner on these nanoscale particles compared to larger pathogens, like bacteria. The small size of viruses has hindered research for centuries and delayed the initial discovery of viruses as infectious agents until the late 1800s [30]. Characterizing the structure of viruses is often an early objective when an emerging virus becomes apparent. Electron microscopy (EM) has been used successfully to determine the size and shape of many viruses [31,32], but the technique provides little information about the function of viral proteins or the distinct differences amongst viral subsets [33]. Cryo-EM techniques have only recently been used to visualize the HIV-1 envelope glycoprotein at high resolution (10 Å) and more recently enabled the first visualization of the crystal structure of the HIV-1 envelope glycoprotein gp120 [34,35].

**Figure 1:**
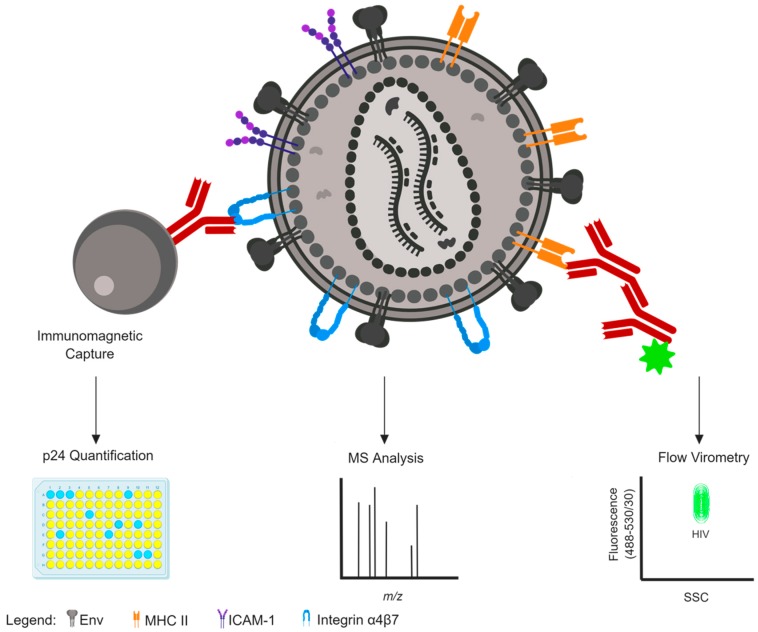
Methods to detect host protein incorporation on the surface of an HIV-1 virion. An HIV-1 particle is depicted with surface antigens displayed, including the viral envelope glycoprotein (Env, grey) and host cell proteins MHC II (orange), CD54/ICAM-1 (purple), and integrin α4β7 (blue). Immunomagnetic bead capture (left) is one common and crude method to determine surface antigen expression on virion surfaces, quantified by p24 antigen ELISA readout. Mass spectrometry (centre) is another common and crude method to detect host protein incorporation that might offer enhanced sensitivity over immunocapture, but cannot distinguish the location of incorporated proteins from virion surface versus virion interior. Flow virometry (right) is an emerging technology for the flow cytometry-based detection of nanoparticles, permitting highly sensitive detection of unique virion populations that can be distinguished based on scattering properties (*x*-axis) and fluorescence expression (*y*-axis) within virions or via staining with fluorochrome-conjugated antibodies (maroon). This figure was created with BioRender.

In enveloped viruses, characterizing the proteins within the envelope is of paramount importance since these proteins often direct infection and tissue tropism and are the primary targets for vaccine design as they are the only antigenic determinants on intact virions that are visible to the immune system [36,37,38,39]. Several techniques have been used to successfully identify and characterize the array of host proteins displayed in viral envelopes. Summaries of some of the more common methods are reviewed below and visually depicted in Figure 1.

### 2.1. Mass Spectrometry

Mass spectrometry (MS) has been a central tool in identifying proteins since the initial development of electrospray ionization (ESI) and matrix-assisted laser desorption/ionization (MALDI) for the probing of intact biomolecules [40]. The ability to determine amino acid composition, molecular mass and post-translational modifications with high sensitivity has greatly expanded the field of proteomics and enhanced our ability to analyze individual proteins and protein complexes [40,41,42]. MS is a common method for determining the total number of proteins associated with a population of viruses [43]. The coupling of MS with other analyses, such as chromatography, has provided many advancements in the field of virology. Liquid chromatography-linked tandem MS (LC-MS) was successfully used to identify the presence of 253 host proteins in HIV-1 virions [28]. Of these proteins, 33 host proteins identified were previously shown to be present in HIV-1 virions using immunocapture techniques (outlined in Section 2.2 below), demonstrating the reliability of MS as a method for detecting host-derived proteins associated with virions [17,44].

Additionally, MS provides the sensitivity to detect viral proteins of low abundance, and conveniently, it can also be used to characterize the impact of viral infection on cellular proteins, while also providing vital information on protein–protein interactions [43]. Comparisons of immunoassays, real-time PCR, and advanced MS techniques (including LC-MS, MALDI-TOF) have found that LC-MS is typically more sensitive than immunoassays [45], while real-time PCR assays were similar in sensitivity to MS [46,47]. Multiple studies using MS techniques have investigated total virion protein contents and successfully identified viral- and host-encoded proteins in vaccinia virus, herpes simplex virus type 1, influenza virus, and HIV-1 [24,25,26,28,48,49,50].

However, while MS is a powerful tool, it does not provide information on the protein composition of individual virus particles, nor does it give any indication on location of the incorporated protein (i.e., intravirion versus virion surface).

### 2.2. Immunomagnetic Capture

Detection of cellular constituents on the HIV-1 envelope are commonly assessed using antibody capture (immunocapture) assays. The sensitivity of antibody detection was used in a seminal study in which MHC class II was first detected on SIV virions through immunoelectron microscopy [51]. Further use of immunoelectron microscopy identified the presence of many other cellular proteins on the surface of HIV-1 virions, including CD11a, CD25, CD54/ICAM-1, CD63, MHC class I and II [13,14]. However, some targets initially identified by immunoelectron microscopy, such as CD4, were unable to be replicated using more sensitive techniques like immunoprecipitation and plate-based monoclonal antibody (MAb) capture [52,53,54,55]. These plate-based MAb assays were employed commonly to capture virions displaying host proteins, followed by p24 (capsid) antigen ELISAs to quantify the amount of virus captured by the antibody [11,52,53]. More recent studies continue to use the principle of the antibody-mediated capture, but instead employ the use of immunomagnetic beads [18,19,29,56,57]. Magnetic bead-based separations permit more reproducible results and lower p24 background levels, and have been able to confirm the presence of previously described host proteins determined through plate-based and immunoelectron microscopy methods [18,54]. The high specificity of immunomagnetic capture assays has been employed to identify differences in the antigenic profile of macrophage- and T cell-derived virions using specific host cell markers exclusive to the respective cell types [56], demonstrating the sensitivity and power of this technique.

One limitation of this technique is that it can only inform on the relative amount of virus precipitated or captured, while no information is gained on the absolute number of host-cell derived proteins on each single virion [58]. The presence of contaminating microvesicles with surface-displayed host antigens can also complicate immunocapture data, although there are techniques to mitigate these effects [59,60]. Pertinent to this review, a list of HIV-incorporated host proteins discovered using the aforementioned assays is shown in Table 1.

### 2.3. Nanoscale Flow Cytometry and Flow Virometry

Flow cytometry is a powerful, well-established tool that is used in clinical and research settings to characterize cells based on light scattering properties and fluorescent emissions [65,66,67,68]. Although flow cytometry can easily characterize single cells, organelles and bacteria, it is difficult to detect smaller particles (below 300 nm in size) using light scattering with traditional cytometers [69,70]. Early work employing a conventional flow cytometer designed to characterize small particles was able to discriminate between reoviruses, T2 bacteriophages, and latex spheres using light scattering [71]. Traditional cytometry methods have also been optimized for marine biology studies seeking to investigate viral populations in water samples [72,73,74]. However, recent and dramatic advancements in techniques have significantly improved the ability to resolve nanoparticles through what has been coined “flow virometry” and more recently “nanoscale flow cytometry” [75,76,77,78,79]. The advancements in this field have been largely based on studies performed on extracellular vesicles (such as exosomes and microvesicles; EVs) which share similar sizes, antigenic profiles and biosynthetic pathways as many enveloped viruses, including retroviruses like HIV-1 [80].

The distinct advantage of flow virometry from bulk methods that are commonly used to study viruses such as western blot, MS or immunocapture, is that it has the power to characterize individual virus particles. The ability to analyze surface antigens on individual virions permits the discrimination between viral subsets that contain varying amounts of host-derived proteins [75]. Furthermore, sorting viral subpopulations allows for specialized studies focusing on how viral characteristics are altered by the incorporation of particular host proteins [70,81]. The similarities in size, density, and antigen profiles between EVs and virions has complicated purification of virion preparations for years [60,82]. However, precise discrimination between EVs and virions can now be successfully achieved with nanoscale flow cytometry techniques [76]. A recent study using both traditional and submicron cytometers indicated that both are adept at detecting individual HIV-1 virions using fluorescence, but not by light scatter [70]. Similar studies using other viruses, including murine leukemia virus (MLV) and vaccinia virus, have successfully visualized viruses using light scattering [76,77]; however, not all instruments are sensitive enough to discriminate between viruses and instrument noise. Additionally, detection through light scattering can lead to increased abort rates [76], confounding data acquisition. It is anticipated that discriminating between viral subsets solely on light scattering properties will become more common as technology improves.

One major difficulty that is encountered when performing flow virometry is swarming or coincidental events. Cytometers are designed to ensure that single cells are analyzed by a detector via a single-cell stream [65,68]; however, virions can be several orders of magnitude smaller than cells and may be detected in groups by the cytometer, reducing the accuracy and reliability of sample measurements [79]. Optimism for single-stream virion analysis has been renewed with recent work showing that optimizing sample concentration through high-order titration can minimize coincidental events and offer highly reliable results (Tang et al. [83] *in preparation*).

Another issue with this technique is the availability of nanoparticle controls. Polystyrene beads which are commonly employed as a control for small-sized particles are not accurate for sizing viruses or EVs since their refractive indices and light scattering properties are quite different from synthetic beads [84,85]. Accordingly, the field is currently developing particle standards and developments have been made on the use of fluorescently labelled viruses or EVs as biological positive controls and size standards (Nanoscale Flow Cytometry for Cancer, Infection, & Disease, University of Ottawa, 2018). Similarly, the development of software to approximate the diameter and refractive indices of nanoparticles based on individual instrument parameters and bead standards is also becoming more common [86,87]. Accurate and comprehensive controls, as well as optimization of signal-to-noise ratios are both imperative to ensure confidence in measurements in this up-and-coming field.

## 3. Host Proteins Incorporated on Virion Surfaces

While many host proteins have been identified in the envelope of HIV-1 virions, only a few have been studied in depth. Despite this void in the literature, there is great value in performing these studies as incorporated proteins can play a large role in viral pathogenesis and infection. Although the majority of host proteins are excluded from the envelope of virions during budding, specific MHC isotypes, adhesion proteins, and complement proteins are commonly found embedded in HIV-1 virions (Table 1). The types of proteins that virions acquire are highly specific to host cell type and viral strain; in fact, the antigen profile on monocyte/macrophage- and T cell-derived virions can be quite different. Indeed, virion capture assays targeting discriminatory proteins between these two cell types have showed significant differences in the antigenic profile of their respective progeny virions [56]. Furthermore, the acquisition of host-derived adhesion molecules that are functionally active can provide a distinct, selective advantage to virions during transmission and infection, as discussed herein. This is especially important for HIV-1 since this virus has a relatively low rate of successful transmission [88]. This section will highlight several of these well-studied HIV-incorporated proteins and mention more recently identified host proteins in the HIV-1 membrane that might affect viral pathogenesis.

### 3.1. Major Histocompatibility Complex (MHC)

The incorporation of MHC class I and II antigens in the viral envelope has been well-established for both HIV-1 and the highly related SIV viruses [11,12,13,14,15,52,54,58,64,89]. Early reports indicated that certain isotypes of human lymphocyte antigen (HLA) class II, such as HLA-DP and HLA-DQ, were absent from virions [12,15]. Subsequent work demonstrated that HLA-DP and HLA-DQ were indeed present on some virions, but that incorporation was dependent on the cell type in which virions were derived from [54]. Conversely, HLA-DR has consistently been found at high levels on the surface of HIV-1 virions derived from multiple cell types, despite it not being avidly expressed on host cell membranes [18,54]. These data suggest a mechanism of selective enrichment of HLA-DR incorporation into virion membranes. Notably, the number of host-derived HLA-DR and β-2 microglobulin molecules associated with SIV and HIV-1 virions were found to outnumber the viral envelope glycoproteins [15], additional evidence suggesting that virions are enriched for select host proteins [12,17]. Furthermore, analyses of molecular mass ratios between HLA-DR (or ICAM-1) to the viral p24 (capsid) protein in virions have also suggested that host proteins can outnumber viral proteins in virus fractions [55,60].

While high levels of MHC molecules, including HLA-DR and β-2 microglobulin, are present on virions, microvesicles that co-purify with virions during sucrose density centrifugation can also express these host antigens, which can confound measurements [60]. The level of MHC expression on virions can vary given the type of cell being infected, the viral strain [54] and for clinical samples, donor variability [90]. Similarly, the number of virions and microvesicles produced that display cellular antigens varies with each cell passage, whereas certain HIV-1 proteins, such as viral envelope (glycoprotein 120, gp120), are less commonly associated with microvesicles [60]. Notably, one of the first groups to demonstrate the presence of MHC class II antigens on SIV virions also showed that chronically-infected (SIV and HIV) cell lines had increased expression of MHC class II compared to uninfected control cells [51]. It was later shown that many incorporated cellular proteins, including MHC II antigens, are functional in the viral envelope and can increase virulence (Section 5.3 of this review). Conversely, HIV-1 infection has been associated with a transient decrease in expression of MHC I antigens on monocytic and T lymphocytic cell lines, likely a mechanism used by virions to evade cytotoxic T lymphocyte killing [91,92,93,94].

### 3.2. ICAM-1 and LFA-1

Intercellular adhesion molecules (ICAM) are involved in numerous cellular processes including adhesion and inflammation. They are often exploited as receptors by pathogens, including human rhinoviruses and *Plasmodium falciparum* malaria parasites [95,96]. ICAM-1, a subset of the ICAM family, is the cognate ligand for the lymphocyte function-associated antigen 1 (LFA-1/αLβ2) [97], another cellular adhesion molecule. The interaction between ICAM-1 and LFA-1 is important in T cell activation, migration of T cells to target sites, and pertinent to HIV-1 infection, in the formation of syncytia. Syncytia are a cytopathic phenomenon associated with HIV-1 infection that is characterized by multiple cell fusion events, leading to the formation of giant multinucleated cells which subsequently lyse and release a burst of virions [98]. While syncytium formation was canonically known to involve gp120 and CD4 interactions, it was also shown that the ICAM-LFA interaction can induce syncytium formation, as blocking LFA-1 with a monoclonal antibody caused an attrition of syncytium formation [99]. ICAMs 1–3 and LFA-1 were later confirmed to be involved in this process [100], as well as involved in increasing HIV-1 infectivity (see Section 5.1 of this review). Furthermore, all of these adhesion molecules have been found as constituents of the HIV-1 envelope in virions propagated in peripheral blood mononuclear cells (PBMC) [55,57,64]. Interestingly, an N-terminal synthetic peptide derived from the ICAM-1 sequence inhibited virus replication and syncytium formation in a dose-dependent manner, indicating that the ICAM-derived peptide may bind to LFA-1 on uninfected cells or virions to competitively antagonize natural interactions with functional (full-length) ICAM-1 [101].

Similarly, antibodies directed against the subunits of LFA-1 and ICAM-3 were shown to inhibit syncytium formation, as well as HIV-1 entry and infectivity in T lymphoid (SupT1, CEM) and monocytoid (U937) cell lines, prompting speculations that ICAM is a key mediator of HIV-1 entry [102]. While ICAMs are not co-receptors for HIV-1 entry, the incorporation of host-derived ICAM-1 was shown to enhance HIV-1 infection in T and monocytic cells through enhanced physical interactions with LFA-1 on target cells [19,23]. More detail regarding the biological effects of ICAM incorporation in HIV-1 infection is outlined below in Section 5.1.

### 3.3. Integrin α4β7

Integrin α4β7, the gut-homing receptor present on CD4^+^ T lymphocytes, facilitates gastrointestinal homing through binding to its cognate ligand, mucosal addressin cell adhesion molecule 1 (MAdCAM-1), which is restricted in expression to only gut tissues [103]. Integrin α4β7 has been of recent interest due to its ability to bind the HIV-1 envelope protein gp120 [104], its applications as a marker of CD4^+^ T cell depletion [105], and most recently, its use as a predictor of HIV-1 acquisition and disease progression [106]. Further interest in α4β7 has been piqued by the in vivo effects of anti-α4β7 monoclonal antibody treatments in SIV-challenged macaques, which led to delayed viral transmission [20], decreased viral loads [21], and persistent control of infection, even after withdrawal of anti-α4β7 treatment [22]. We recently showed that HIV-1 virions from clinical and laboratory-adapted isolates, as well as SIV strains, can incorporate α4β7 into their viral membrane and that the integrin remains biologically active when displayed on the surface of virions [18]. Surprisingly, the amount of α4β7 incorporation in viral envelopes was found to be significantly higher than the well-characterized ICAM-1, LFA-1, HLA-DR, and CD43, although the latter two did not reach statistical significance [18]. The marked enrichment of integrin α4β7 on HIV-1 virions strongly suggests a selective mechanism of incorporation. While this mechanism has not yet been fully elucidated, it is suspected to be Gag-dependent, similar to that for ICAM-1 incorporation [29]. The incorporation of integrin α4β7 into virions was also shown to be relevant in clinical disease progression. Indeed, high levels of virion-incorporated α4β7 were detected in sera from patients during acute HIV-1 (and SIV-1) infection [18], which is in accordance with high levels of viral replication in α4β7+ intestinal CD4^+^ T cells during this acute phase [107,108,109].

### 3.4. Complement Proteins

The complement system consists of over 30 soluble and cell surface proteins that bridge the innate and adaptive immune systems. The complement pathway leads to increased opsonization [110] and/or direct lysis of pathogens through the formation of a membrane-attack complex [111]. Notably, HIV-1 infection has been associated with modified expression of complement proteins on the surface of erythrocytes and infected T cells [112,113]. However, while HIV-1 virions activate complement, they are not readily neutralized by this host defense mechanism [114]. Complement proteins, such as the glycosylphosphatidylinositol (GPI)-linked proteins CD55 and CD59, which are canonically anchored to the cell membrane, have been found to be displayed on HIV-1 virion surfaces [115]. Furthermore, virions produced in cell types that lacked CD55 and CD59 were shown to be sensitive to complement-mediated neutralization, whereas virions produced in cells that expressed the proteins were found to be resistant to this host defense [115]. Both of these proteins, in addition to CD46, a complement regulatory protein, have also been found within SIV virions [53]. Interestingly, blocking these proteins with targeted antibodies resulted in increased virion lysis as well as a reduction in infectivity for both HIV-1 and SIV [53].

### 3.5. Other Molecules

It is noteworthy that the presence of many other adhesion molecules such as CD44, CD63 (tetraspanin), and CD62L (l-selectin) has been detected in the HIV-1 envelope [11,63,64]. However, the effects that these host molecules have on viral pathogenesis have not been well-characterized and it is likely that additional undescribed host proteins are also present in the viral envelope [28].

Until recently, CD40 and its cognate ligand CD40L (CD154), a receptor/counter-receptor pair that are involved in humoral immunity and tolerance [116,117], were not well-established in the HIV-1 envelope [62]. While it was known that CD40L is expressed on CD4^+^ T cells and that CD40 is expressed on APCs, both antigens were found to be incorporated in the viral envelope in viral strains of CXCR4- and CCR5-coreceptor usage in PBMCs and tonsillar tissue [61]. Further studies revealed that CD40L remains functionally active in virions and is able to mediate NF-κB activation [118] and induce immunoglobulin G (IgG) and interleukin-6 production [119]. These findings and others suggest that virion-incorporation of CD40L may play a role in B cell abnormalities associated with HIV-1 infection [119]. Taken together, an overwhelming amount of evidence supports the idea that many host-derived proteins remain functionality active in virions, with the ability to affect viral pathogenesis and tropism (further discussed in Section 5).

## 4. Mechanisms of Incorporation

Early experiments that screened HIV-1 virions for the incorporation of host proteins noted that specific proteins, such as CD4, CD80, and CD87, were commonly excluded from the outer envelope [52,55,58] of virions, while other proteins like MHC class II (e.g., HLA-DR) seemed selectively enriched [11,12]. Although HIV-1 has a small genome, it has many mechanisms in place to avoid host defenses and it likely has also evolved mechanisms to selectively uptake antigens from its host cell to increase infectivity. Indeed, the level of protein expression on a cell surface does not necessarily correlate with the virion-incorporated amount of the same protein [11,12,15,18,120], indicating that incorporation is not simply a passive uptake of antigens, but instead a selective mechanism of uptake. Further evidence of selective incorporation is the select panel of conserved antigens on HIV-1 and SIV virions, even when different cell types are used to propagate the viruses [15,18,59,121,122]. Detailed below are potential mechanisms through which HIV-1 could acquire its unique antigenic profile and examples of studies performed to elucidate the viral determinants associated with virion-incorporation of MHC class II and ICAM-1.

### 4.1. Budding Through Lipid Rafts

While there are distinct differences between the antigenic profiles on T cell-derived and macrophage-derived viruses, 544 individual proteins (79 protein clusters) were described as shared between the two viruses after a quantitative proteomic study was performed [59]. The antigenic similarities suggest a common egress pathway that facilitates acquisition of select host proteins [59]. One mechanism by which this may be occurring is via selective budding through lipid rafts [123,124].

Lipid rafts are small, mobile sections within membranes that are detergent-resistant and enriched with cholesterol, sphingolipids, and GPI-linked proteins [125,126], including the complement proteins CD55 and CD59. These rafts have been implicated in multiple aspects of the HIV-1 replication cycle, including viral entry and egress [123,127,128]. The involvement of lipid rafts during viral egress has also been noted in other enveloped viruses such as influenza virus, Ebola virus, and measles virus [128]. Notably, the HIV-1 viral membrane is also enriched in cholesterol and sphingolipids [129], displaying areas reminiscent of cellular lipid rafts, and this lipid composition in the viral membrane has been shown to have implications for infectivity [130,131,132]. Specific depletion of cholesterol in T cell lines and HIV-1 virions were both shown to decrease infection [130,132,133], suggesting the importance of raft integrity in viral infection. However, cholesterol may not play a significant role in mediating host protein incorporation into virions, since destabilizing it with chemical treatments did not affect the association of host proteins (MHC I and II) with the viral envelope [132].

While the lipid composition [134] and the incorporation of the GPI-anchored lipid raft markers within virions both support the suggestion that incorporation into HIV-1 mechanistically occurs via budding through lipid rafts, it is clear that other viral determinants must be involved in the acquisition of host proteins by HIV-1 virions. For example, CD45 and CD14 are both resident within lipid rafts in T cells [135,136] and macrophages [120], but both proteins are seemingly excluded from virion envelopes or present at low levels [11,120,123]. This specific exclusion is especially notable since cellular membranes contain high levels of CD45, while virions do not [58,137]. It has been suggested that the exclusion of CD45 could simply be due to steric hinderance [138], but it is probable that HIV-1 has sophisticated mechanisms in place that block the uptake of specific proteins. Similarly, CD4 and CD48 are both well-established lipid raft components which are known to colocalize with raft-resident markers [133,139,140,141,142]; however, HIV-1 has been shown to acquire CD48 [52] while CD4 is excluded from the HIV-1 envelope [52,53,54,55]. It should be noted however, that the absence of CD4 on virions is likely impacted by the low expression of CD4 on infected cells, mediated through HIV-1 Nef- and Vpu-dependent mechanisms that respectively downregulate CD4 expression via endocytosis [143] and degradation of CD4 [144].

While there is a body of evidence supporting the critical role of lipid rafts, it is likely that other mechanisms support the acquisition of select host proteins into virion membranes. For example, the enveloped rhabdoviruses and alphaviruses can contain host GPI-anchored proteins and cholesterols that are not associated with lipid rafts, and similarly, host proteins that are not lipid raft-associated such as the transferrin receptor (CD71), have also been described in HIV-1 virions [11,52,145,146].

### 4.2. Assembly Within Exosomes

Although macrophage-derived virions can also acquire lipid raft-associated markers, including CD55 and CD36 [120], a number of studies have shown that the host protein composition of T cell-derived virions is quite different from macrophage-derived virions [28,59], likely due to the differences between the HIV-1 infectious cycles in T cells versus macrophages. The use of the endocytic pathway, which is more common in monocyte/macrophage infection, permits HIV-1 virions to be taken up and harbored within multivesicular endosomes [147] (also termed multivesicular bodies [120,148]). The fate of virions trafficking through an endocytic pathway is notable, in that these viruses can subsequently exit the cell via an egress pathway similar to the traditional exosome pathway [120,149,150]. Thus, we infer that viruses leaving their host cells via exosomal pathways will likely have a distinct set of incorporated host proteins compared to those leaving through canonical budding pathways.

Exosomes contain features similar to virions, including an enrichment of cholesterol and sphingolipids and an array of cellular proteins such as MHC class I and class II, integrin α4, CD45, CD63, and lysosomal-associated membrane protein 1 (LAMP-1) [120,151]. In monocyte-derived macrophages (MDMs) the late endosome can be a site of HIV-1 assembly [152,153], likely supporting the antigenic similarities between surfaces of HIV-1 virions and exosomes [154]. Gould et al. have posited the “Trojan exosome hypothesis”, suggesting that retroviruses can exploit the cellular vesicle trafficking network for both the production of new virions and the transmission of infectious particles via exosomal uptake in neighboring uninfected cells [150]. Taken together, these studies could help explain the different protein composition observed in T cell-derived and macrophage-derived viruses, and may also explain why macrophage-derived exosomes are similar in their host protein profile to HIV-1 particles derived from the same parent (host) cells [120].

However, while the presence of virions within intracellular vacuoles is widely accepted [155], more robust empirical evidence is still required to fully substantiate the entirety of the Trojan exosome hypothesis [154]. Regardless of this, the similarities between HIV-1 and exosomes are well-established and further studies may help clarify the role of exosomes in HIV’s acquisition of host proteins.

### 4.3. Mechanisms Involving Cytoskeletal Proteins

Cytoskeletal proteins are essential for a wide array of cellular processes and unsurprisingly, they have been implicated in many steps of the HIV replication cycle [156]. Since HIV has mechanisms in place to hijack many aspects of the cytoskeleton, it is possible that it may also modulate the cytoskeleton to selectively uptake specific proteins. To initiate infection, HIV-1 can enrich the presence of its co-receptors and CD4 to productively fuse with its target cells. To do this, HIV-1 triggers signaling cascades within cells that induce actin-dependent clustering of entry receptors through the recruitment of filamin A, a protein which serves as a link between the entry receptors and actin [156,157,158,159]. It is possible that similar signaling cascades may be utilized by virions to trigger the clustering of host proteins that are selectively acquired by budding virions.

Notably, studies have shown that disruptive drug treatments which target motor proteins, the actin network and/or GTP-binding proteins can hinder or block viral budding [160,161]. Similarly, the HIV-1 Gag protein is described to associate with actin and the microtubule motor kinesin member 4 [160,162,163,164], which may suggest a role for cytoskeletal proteins in virus egress [165]. While further studies will need to be conducted to discriminate the exact role of cytoskeletal proteins in viral budding, remodeling of actin has been documented at budding sites [158]. Manipulation of actin-dependent pathways to impact viral replication has recently been shown as a mechanism employed by the anti-viral cytokine interleukin-27 (IL-27). A new mechanism underlying the anti-HIV-1 activity of IL-27 was shown to be the downregulation of spectrin beta nonerythrocyte 1 (SPTBN1), a newly described host factor that is required to support HIV-1 infection in monocytes and macrophages [166]. Notably, the SPTBN1 protein is involved in binding actin to the plasma membrane [167] and has been demonstrated to associate directly with HIV-1 Gag [166]. The reliance on SPTBN1 for successful HIV-1 infection was demonstrated by SPTBN1 knockdown which conferred HIV-resistance in macrophages, while overexpression of SPTBN1 rendered macrophages sensitive to infection [166]. Furthermore, the HIV accessory protein Nef is known to modulate host actin levels [156,168,169], and therefore, it may be informative to probe the role of Nef in future studies aimed at delineating the viral determinants of host protein incorporation. Although a concrete link is yet to be established between the cytoskeletal arrangement and the acquisition of host proteins into virions, additional studies are highly merited to discern the connections between these two phenomena.

### 4.4. Mechanisms of MHC and ICAM-1 Incorporation

Mechanistic studies of MHC class II (HLA-DR) incorporation found that neither the presence of the viral envelope glycoprotein (gp120) or Nef accessory protein affected the incorporation of HLA-DR [170], with similar results observed for ICAM-1 incorporation [171]. However, another study that employed whole virus immunoprecipitation with patient antisera observed that virion-incorporation of MHC class II distinctly required the envelope glycoprotein, while this was not the case for MHC I proteins [172]. It is likely that these conflicting results may be due to differences in sensitivity of experimental assays. More recently, a detailed study confirmed the dispensable role of gp120, and instead supported a role for the HIV structural protein Gag (matrix domain) in the incorporation of ICAM-1 [29]. Assays employing a virus-like-particle (VLP) system, three-dimensional modeling and amino acid substitution mutants, determined that the HIV matrix protein (p17) is required for ICAM-1 incorporation into both VLPs and infectious virus. With these studies we acknowledge that it is also possible that the divergent results on the role of HIV envelope (gp120) may be indicative that MHC II and ICAM-1 are incorporated via distinct molecular mechanisms, employing unique viral determinants.

## 5. Effects of Host Protein Incorporation on Viral Pathogenesis

A large body of work supports the role of adhesion, integrin, complement, and MHC proteins in HIV-1 pathogenesis [18,23,115,119,173,174,175]. For example, specific complement proteins incorporated into the viral envelope can help virions avoid complement-mediated lysis [115] while integrins and other adhesion molecules like ICAM-1 can retain their functionality and bind their cognate ligands to increase infectivity [18,19]. However, the advantages (and disadvantages) that viruses are afforded from incorporating host proteins is highly dependent on tissue tropism, route of infection, and mechanisms of in vivo virus trafficking [18,54].

### 5.1. ICAM-1

Strikingly, HIV-1 virions that display ICAM-1 in their outer membrane were found to be more infectious (2- to 9-fold) than virions without ICAM-1 [19,23], and activation of cellular LFA-1 enhanced infection with ICAM-bearing viruses by over forty-fold in both cell lines and PBMC [173,176]. A more clinically relevant study using ex vivo human tonsillar tissue corroborated the importance of ICAM-1 in increasing infection, whereby the advantages gained by virions bearing functionally active ICAM-1 were negated through the use of an anti-ICAM monoclonal antibody [176]. Furthermore, CD4^+^ T cell depletion was found to be more profound upon infection with ICAM-positive virions [176]. Notably, ICAM-containing virions showed increased resistance to antibody-mediated neutralization with anti-gp120s [23], while treatment with an anti-ICAM-1 antibody dramatically reduced the entry efficiency of ICAM-bearing virions by ~100-fold [23]. These data align with other studies that have shown that the ICAM-LFA interaction can facilitate infection in CD4^+^ T cells [173,174]. Additionally, the ability of anti-gp120 monoclonal antibodies to neutralize ICAM-positive virions was decreased when cells were treated with an antibody which activated its cognate ligand, LFA-1 [174]. Similarly, compared to virions devoid of ICAM-1, ICAM-1-positive virions demonstrated an increased resistance to neutralization using human sera from HIV-infected individuals if the target cells also expressed LFA-1 [174]. ICAM-1 and LFA-1 have also been implicated in adhesion of HIV-1 virions to follicular dendritic cells (FDCs) [177]. The capture and spread of HIV-1 to FDCs is of importance because while FDCs are not permissive to infection, they can carry infectious virions [178] and transfer them to bystander CD4^+^ T cells. Furthermore, ICAM-LFA interactions may also fuel infectivity by mediating viral synapses and immunological synapses to increase the efficiency of cell-to-cell transmission [19,99,173,174,179]. Thus, while the importance of ICAM-1 in HIV-1 infection is notable, the effects of its cognate ligand, LFA-1, are also highly significant, as it is involved in mediating viral and immunological synapses that can increase the efficiency of cell-to-cell transmission. Based on this, it is likely that the two proteins play important roles during infection in vivo that have not yet been described.

### 5.2. Other Adhesion Proteins and Integrins

The incorporation of leukocyte L-selectin (CD62L) into HIV-1 envelopes has been previously been established [63,64]. Indeed, CD62L is functionally active in virions and enhances adsorption of virions to endothelial cells, resulting in increased virus transmission to CD4^+^ T cells [63]. The interaction of virion-incorporated host-derived proteins with their cognate physiological receptors can be important mediators of trans-infection and can have significant influences on in vivo virus homing. For example, a recent study corroborated this importance with HIV-1 viruses that have incorporated the principle gut-homing integrin, integrin α4β7. As a proof of concept for trans-infection, the α4β7-positive virions were shown to be efficiently captured by non-permissive cells expressing the cognate ligand MAdCAM-1, retained after extensive washing, and readily transferred to overlayed target cells [18]. This study also employed an in vivo animal model to show that HIV-1 viruses bearing surface-displayed α4β7 homed rapidly and selectively to gut tissues, driven by interaction with their cognate ligand MAdCAM-1, which has restricted expression to gut-resident endothelial cells. Thus, while the MAdCAM-1 that lines endothelial cells canonically functions to draw circulating α4β7^+^ CD4^+^ T cells to the gut mucosae, recent evidence indicates that viruses can hijack such mechanisms of cellular homing, resulting in more efficient transit to hot-spots of permissive cells, likely fueling viral pathogenesis in the long run [18].

### 5.3. Major Histocompatibility Complex (MHC)

Both MHC class I and II molecules have been found associated with HIV-1 membranes and are known to affect viral pathogenesis [149]. The human MHC isotype HLA-DR has been reported to be one of the most abundant host proteins within virions and its effects on pathogenesis have been studied closely. The acquisition of MHC II is highly notable since CD4 is the cognate ligand of MHC and early studies demonstrated an interaction between the two: MHC on virions and CD4 on cell surfaces [180]. More specifically, 293T cells that were transfected with HLA-DR1 genes produced virions with membrane bound HLA-DR1; these virions were shown to have increased infectivity in T cell and monocytic lines, as well as in PBMC [180]. The incorporation of HLA-DR1 also increased the kinetics of infection [180]. Further work done in a B cell line permissive to infection and bearing MHC II corroborated previous work by showing that MHC II-positive virions were more efficient at infecting several T cell lines than MHC II-negative virions and that this infection occurred more rapidly [181].

A study that aimed to test the functionality of HLA-DR in antigen presentation showed that virions with incorporated HLA-DR, but not those devoid of HLA-DR, could interact with a bacterial toxin (staphylococcal enterotoxin A) to stimulate the proliferation of T cells and induce the production of interleukin-2 (IL-2) [182]. This suggests that HLA antigens maintain some kind of functional role even when virion-bound, similar to other incorporated proteins. Indeed, further evidence for this suggestion was that HLA-DR-positive virions displaying the bacterial toxin were also able to induce T cell apoptosis [182]. However, the authors did note that other viral accessory proteins may have played an undescribed role in this phenomenon.

The effect of human MHC class I (HLA I) incorporation on HIV-1 infection has also been investigated. Indeed, the infectivity of both laboratory strains and primary HIV-1 isolates was increased upon virion incorporation of HLA I, and a lower susceptibility to antibody-mediated neutralization with seropositive sera was also observed upon HLA I incorporation into virions [175]. Taken together, these studies provide compelling evidence for the role of both MHC I and II incorporated antigens in HIV-1 pathogenesis. Future in vivo studies are warranted to help provide insight on the role of these incorporated proteins in HIV disease progression.

## 6. Novel Targets and Treatments

Although many advancements in antiviral treatments have been made since the discovery of HIV as the etiologic agent of AIDS, no cure has been elucidated to date. While progress on pre-exposure prophylaxis and antiretroviral drugs has greatly reduced the risk of HIV transmission and improved the lives of individuals living with HIV, the costs, adverse effects, and inconvenience of lifetime treatments make these therapies undesirable. Most recently, a large interest in monoclonal antibody therapies and latency reversing agents has become the major focus in the field [183]. Given the current interest in alternative therapeutics, host-derived proteins on HIV-1 surfaces provide an attractive and novel repertoire of antigenic targets to be considered in new therapeutic and vaccine designs. The following section highlights host-derived targets on virions that are currently being explored or employed in anti-HIV treatments.

### 6.1. Therapeutic Targeting of Integrin α4β7

The envelope glycoprotein (gp120) of certain HIV isolates has been shown to bind to α4β7 [104,184], although this finding is not reproducible among all gp120s assessed [185]. Targeting α4β7 in activated primary CD4^+^ T cells with both an anti-α4β7 antibody and short hairpin RNAs led to a decrease in HIV-1 infection [184], although the former may be confounded by cell aggregation which can be induced by anti-α4β7 antibodies. A series of macaque studies employing the anti-α4β7 monoclonal antibody, ACT-1, showed that the antibody could limit viral replication in acutely-infected animals [21,186], and protect uninfected animals in transmission studies with repeated mucosal challenge [20]. Most striking were the recent macaque studies showing that combined treatment with antiretroviral therapy and primatized anti-α4β7 antibody elicited sustained suppression of viral replication, even after both therapies were removed [22]. Given the range of recent macaque studies, it is evident that α4β7 may be important target in in vivo HIV infection in humans as well. Furthermore, the expression of α4β7 on human CD4^+^ T cells has been associated with increased HIV-1 acquisition, increased CD4^+^ T cell decline, and higher set-point viral load [106]. It is notable that the same anti-α4β7 antibody used in the various macaque studies is also an approved treatment for irritable bowel disease patients (ulcerative colitis and Crohn’s disease), currently manufactured as a humanized antibody under the name Vedolizumab (Takeda Pharmaceuticals). Indeed, a recent study of HIV-infected patients being treated for inflammatory bowel disease with Vedolizumab demonstrated that the treatment was safe and caused a reduction in lymphoid aggregates [187]. While this study indicates that Vedolizumab may help reduce viral gut reservoirs, further studies are warranted and ongoing to determine if Vedolizumab is an efficacious anti-HIV treatment option. A clinical trial aimed to evaluate the effect of Vedolizumab in HIV-infected individuals is ongoing and is scheduled for completion in 2020 (see ClinicalTrials.gov, Identifier: NCT02788175). Clinical trials using Vedolizumab for HIV-1 treatment are also underway in Spain and Canada (NCT03577782, NCT03147859), and planned in France (NCT02972450).

### 6.2. Therapeutic Targeting of MHC Proteins

An efficacious HIV vaccine, which is likely a requirement for global HIV eradication [188], has been researched for decades and to date remains one of the greatest challenges in modern medicine. The primary target for canonical HIV vaccines is the envelope glycoprotein, as it is the only viral protein visible on the surface of intact virions to the immune system [38]. However, due to the high error rate of reverse transcriptase, mutations often occur during viral replication cycles, resulting in evasion of antibody recognition and cell-mediated responses, and antiviral drug resistance [189]. Moreover, the relatively low level of envelope glycoprotein expression on virion surfaces further hinders vaccine efficacy. MHC class II antigens are frequently incorporated into HIV virions and are much more abundant than envelope glycoproteins [11,12,13,15,18], thus, these antigens may provide additional attractive targets in HIV vaccine design [190,191,192]. This idea was highly favourable in early years [191] and has continued to be an active area of research [192,193,194].

Numerous reports have suggested that alloimmunization with MHC antigens can elicit protection in a myriad of ways, and may be correlated with a reduction in HIV-1 transmission [195,196,197]. Indeed, the use of an alloimmune MHC (HLA) vaccine has been pursued with decades of study by many different research groups. One study based on World Health Organization data from 38 different African countries identified a negative correlation between HIV infection rates and ethnic diversity [198]. Given that increased ethnic diversity is associated with increased MHC discordance, the authors suggested that this discordance may help suppress HIV-1 transmission and that an MHC-based alloimmunization strategy could lead to an alloimmune response that may be more efficacious than traditional vaccine designs [198]. While traditional vaccine design focuses on generating protective immune responses based on viral epitopes, an MHC-based alloimmune vaccine considers viral (i.e., gp120) proteins together with an emphasis on foreign host proteins (i.e., MHC) that can elicit an alloimmune response. For example, when HIV spreads to a new host, it carries the MHC antigens derived from its former host which new recipients can target with an alloimmune response. However, this activity is lost after infection within the new host occurs and progeny virions acquire the autologous MHC signature of the new host [198].

Early macaque studies supported the efficacy of MHC-based alloimmunization strategies; macaques that were challenged with SIV after being immunized with inactivated SIV produced in human T cell lines experienced complete protection in the majority of the immunized animals [199,200,201,202]. In these studies, the vaccine-elicited protection was attributed to the allogeneic MHC II antigens that the virions carried from human cells [190] and vaccine designs based on this principle have continued to be pursued [193,194]. However, it should be noted that while the benefits of alloimmunization have proven protective in SIV studies, alloantigens such as MHC molecules, as well as other incorporated host antigens such as ICAM-1 or LFA-1, can maintain their biological function on virion surfaces, potentially facilitating interaction with and infection of CD4^+^ T cells [19,203].

Notably, other vaccines that have targeted host proteins have also proven to be beneficial in select animal models. As noted previously, both CD40 and CD40L can be incorporated into virions and this incorporation may have direct implications on aberrant immune responses during HIV-1 infection [61]. One study performed in a humanized-mouse model has shown that a therapeutic CD40-targeting HIV-1 vaccine paired with a TLR3 agonist can induce HIV-specific T cell responses [204]. The vaccine was also able to reactivate and reduce the levels of HIV-1 DNA in latent reservoirs within lymphoid tissues, as well as delay viral rebound upon removal of antiretroviral treatments [204]. Though these in vivo results are likely due to a myriad of factors that will be challenging to discriminate, the role of virion-associated host proteins may play a significant part in this vaccine-elicited protective response.

While a large body of work gives credibility to the idea of an MHC alloimmunization vaccine design, the search for an HIV vaccine is still ongoing and far from realization. Despite the many hurdles, the benefits of targeting host proteins embedded in virions that are more readily accessible and available than the highly variable envelope glycoproteins may be promising in the design of an efficacious HIV vaccine. 

## 7. Conclusions

The incorporation of biologically active host proteins into animal lentiviruses during the budding process is selective and conserved, and in many cases, can induce increased viral pathogenesis. Although the detailed effects of only a few host proteins on viral pathogenesis have been characterized, the recent emergence of the highly sensitive technique of nanoscale flow cytometry (flow virometry) may allow for easier identification of host proteins on individual virus subsets, and sorting of these subsets to determine the impacts of incorporated proteins on viral infectivity and pathogenesis. Given the vast number of host proteins acquired on the surface of enveloped virions like HIV-1, it is likely that additional studies on these proteins may provide valuable new information that could inform new therapeutics and vaccine designs.

## Figures and Tables

**Table 1:** Host proteins incorporated into the external HIV-1 envelope.

Host Protein	Host Cell	HIV Isolate	Method of Detection	Reference
CD3	H9, CD4+ T cells, Monocytes, PBMC	Ba-L, f/s.8, IIIB	EM, PB-cap, IM-cap	[13,52,56]
CD5	H9, M8166, PBMC	IIIB	EM, PB-cap	[13,52]
CD6	H9, M8166	IIIB	PB-cap	[52]
CD11a/CD18 (LFA-1)	PBMC, SupT1, CEMX174, Jurkat, M8166, U937, H9, C8166	IIIB, SF162, HTLV-IIIRF, LAI	PB-cap, IM-cap	[11,14,18,52,55]
CD11b/CD18 (Mac1)	U937, M8166	IIIB	PB-cap	[52]
CD11c (integrin αX)	U937, M8166, PBMC	IIIB	PB-cap	[52]
CD25	CD4+ T cells, Monocytes	Ba-L, f/s.8	EM, IM-cap	[13,14,56]
CD26	CD4+ T cells, Monocytes	Ba-L, f/s.8	IM-cap	[56]
CD27	PBMC	SF162	IM-cap	[18]
CD29 (integrin β1)	PBMC, H9, U937	IIIB	IM-cap, PB-cap	[18,52]
CD30	H9	IIIB	EM	[13]
CD36	CD4+ T cells, Monocytes	Ba-L, f/s.8	IM-cap	[56]
CD40/CD40L	DC, PBMC	Ba-L, CI	IM-cap, PB-cap	[61,62]
CD43	PBMC, U937, SupT1, CEM.NK^r^, Jurkat, H9	IIIB, SF162, HTLV-IIIRF	IM-cap, PB-cap	[11,18,52]
CD44	PBMC, CD4+ T cells, Monocytes, SupT1, CEM.NK^r^, CEMX174	Ba-L, f/s.8	IM-cap, PB-cap	[11,56]
CD45	PBMC, Jurkat	SF162, HTLV-IIIRF	IM-cap, PB-cap	[11,18]
CD46	PBMC, CEMX174	SF162, IIIB	IM-cap, IP	[18,53]
CD48	PBMC, U937, H9, M8166	IIIB	PB-cap	[52]
CD49d (integrin α4)	PBMC	SF162	IM-cap	[18]
CD54 (ICAM-1)	PBMC, U937, H9, M8166, C8166	IIIB, SF162, LAI, CI	EM, IM-cap, PB-cap	[14,18,52,54,55]
CD55	CEMX174, PBMC, U937, H9, M8166	IIIB	IP, PB-cap	[52,53]
CD59	CEMX174, PBMC, H9, M8166	IIIB	IP, PB-cap	[52,53]
CD62L (L-selectin)	CD4+ T cells, CEM-SS, PBMC	NL4-3, IIIB	IM-cap, PB-cap	[63,64]
CD63 (tetraspanin)	SupT1, CEM. NK^r^, Jurkat, H9, PBMC	IIIB, HTLV-IIIRF	PB-cap, EM	[11,13,14]
CD64	CD4+ T cells, Monocytes	Ba-L, f/s.8	IM-cap	[56]
CD71 (transferrin receptor)	H9, SupT1, CEMX174, PBMC, U937	IIIB, HTLV-IIIRF	PB-cap	[11,52]
CD102 (ICAM-2)	PBMC	SF162	IM-cap	[18]
CDw108 (semaphorin 7A)	H9, U937, M8166	IIIB	PB-cap	[52]
MHC Class I:				
HLA-ABC	H9, PBMC, U937, M8166	IIIB, SF162, HTLVIII, CI	EM, IM-cap, PB-cap	[12,14,18,52,54]
β-2 microglobulin	H9, C8166	IIIB, HTLVIII, CI	PB-cap	[12,55]
MHC Class II:				
HLA-DR, -DP, -DQ	PBMC, H9, U937, M8166, H9, HUT 78, Molt 4 clone 8, C8166	HTLV-IIIRF, SF162, IIIB, LAI, NL4-3, CI	EM, IM-cap, PB-cap, WB	[11,12,13,18,52,54,55]
Integrin α4β7	PBMC	Ba-L, IIIB, SF162	IM-cap	[18]

IM-cap: Immunomagnetic bead capture; PB-cap: Plate-based capture; EM: Immunoelectron microscopy; WB: Western blot; IP: Immunoprecipitation; CI: Clinical isolates.

## References

[1] Chandran K. (2005). Endosomal Proteolysis of the Ebola Virus Glycoprotein Is Necessary for Infection. Science.

[2] Smith G.J., Vijaykrishna D., Bahl J., Lycett S.J., Worobey M., Pybus O.G., Ma S.K., Cheung C.L., Raghwani J., Bhatt S. (2009). Origins and evolutionary genomics of the 2009 swine-origin H1N1 influenza A epidemic. Nature.

[3] Chan D.C., Fass D., Berger J.M., Kim P.S. (1997). Core Structure of gp41 from the HIV Envelope Glycoprotein. Cell.

[4] Chen B.J., Lamb R.A. (2008). Mechanisms for enveloped virus budding: Can some viruses do without an ESCRT?. Virology.

[5] Garoff H., Hewson R., Opstelten D.-J.E. (1998). Virus maturation by budding. Microbiol. Mol. Biol. Rev..

[6] Schols D., Pauwels R., Desmyter J., De Clercq E. (1990). Dextran sulfate and other polyanionic anti-HIV compounds specifically interact with the viral gp120 glycoprotein expressed by T-cells persistently infected with HIV-1. Virology.

[7] Novikoff A.B., Beard D., Beard J.W. (1962). Electron Microscopic Study of the Atpase Activity of the Bai Strain a (Myeloblastosis) Avian Tumor Virus. J. Cell Biol..

[8] Aoki T., Stephenson J.R., Aaronson S.A. (1973). Demonstration of a Cell-Surface Antigen Associated with Murine Sarcoma Virus by Immunoelectron Microscopy. Proc. Natl. Acad. Sci. USA.

[9] De-Thé G. (1964). Cytoplasmic Microtubules in Different Animal Cells. J. Cell Biol..

[10] Azocar J., Essex M. (1979). Incorporation of HLA antigens into the envelope of RNA tumor viruses grown in human cells. Cancer Res..

[11] Orentas R.J., Hildreth J.E.K. (1993). Association of Host Cell Surface Adhesion Receptors and Other Membrane Proteins with HIV and SIV. AIDS Res. Hum. Retrovir..

[12] Hoxie J.A., Fitzharris T.P., Youngbar P.R., Matthews D.M., Rackowski J.L., Radka S.F. (1987). Nonrandom association of cellular antigens with HTLV-III virions. Hum. Immunol..

[13] Meerloo T., Parmentier H.K., Osterhaus A.D.M.E., Goudsmit J., Schuurman H.-J. (1992). Modulation of cell surface molecules during HIV-1 infection of H9 cells. An immunoelectron microscopic study. AIDS.

[14] Meerloo T., Sheikh M.A., Bloem A.C., de Ronde A., Schutten M., van Els C.A.C., Roholl P.J.M., Joling P., Goudsmit J., Schuurman H.-J. (1993). Host cell membrane proteins on human immunodeficiency virus type 1 after in vitro infection of H9 cells and blood mononuclear cells. An immuno-electron microscopic study. J. Gen. Virol..

[15] Arthur L.O., Bess J.W., Sowder R.C., Benveniste R.E., Mann D.L., Chermann J.-C., Henderson L.E. (1992). Cellular proteins bound to immunodeficiency viruses: Implications for pathogenesis and vaccines. Science.

[16] Ott D.E. (1997). Cellular proteins in HIV virions. Rev. Med. Virol..

[17] Tremblay M.J., Fortin J.-F., Cantin R. (1998). The acquisition of host-encoded proteins by nascent HIV-1. Immunol. Today.

[18] Guzzo C., Ichikawa D., Park C., Phillips D., Liu Q., Zhang P., Kwon A., Miao H., Lu J., Rehm C. (2017). Virion incorporation of integrin α4β7 facilitates HIV-1 infection and intestinal homing. Sci. Immunol..

[19] Fortin J.-F., Cantin R., Lamontagne G., Tremblay M. (1997). Host-derived ICAM-1 glycoproteins incorporated on human immunodeficiency virus type 1 are biologically active and enhance viral infectivity. J. Virol..

[20] Byrareddy S.N., Kallam B., Arthos J., Cicala C., Nawaz F., Hiatt J., Kersh E.N., McNicholl J.M., Hanson D., Reimann K.A. (2014). Targeting α4β7 integrin reduces mucosal transmission of SIV and protects GALT from infection. Nat. Med..

[21] Ansari A.A., Reimann K.A., Mayne A.E., Takahashi Y., Stephenson S.T., Wang R., Wang X., Li J., Price A.A., Little D.M. (2011). Blocking of α4β7 Gut-Homing Integrin during Acute Infection Leads to Decreased Plasma and Gastrointestinal Tissue Viral Loads in Simian Immunodeficiency Virus-Infected Rhesus Macaques. J. Immunol..

[22] Byrareddy S.N., Arthos J., Cicala C., Villinger F., Ortiz K.T., Little D., Sidell N., Kane M.A., Yu J., Jones J.W. (2016). Sustained virologic control in SIV+ macaques after antiretroviral and α4β7 antibody therapy. Science.

[23] Rizzuto C.D., Sodroski J.G. (1997). Contribution of virion ICAM-1 to human immunodeficiency virus infectivity and sensitivity to neutralization. J. Virol..

[24] Chung C.-S., Chen C.-H., Ho M.-Y., Huang C.-Y., Liao C.-L., Chang W. (2006). Vaccinia Virus Proteome: Identification of Proteins in Vaccinia Virus Intracellular Mature Virion Particles. J. Virol..

[25] Resch W., Hixson K.K., Moore R.J., Lipton M.S., Moss B. (2007). Protein composition of the vaccinia virus mature virion. Virology.

[26] Yoder J.D., Chen T.S., Gagnier C.R., Vemulapalli S., Maier C.S., Hruby D.E. (2006). Pox proteomics: Mass spectrometry analysis and identification of Vaccinia virion proteins. Virol. J..

[27] Stegen C., Yakova Y., Henaff D., Nadjar J., Duron J., Lippé R. (2013). Analysis of Virion-Incorporated Host Proteins Required for Herpes Simplex Virus Type 1 Infection through a RNA Interference Screen. PLoS ONE.

[28] Chertova E., Chertov O., Coren L.V., Roser J.D., Trubey C.M., Bess J.W., Sowder R.C., Barsov E., Hood B.L., Fisher R.J. (2006). Proteomic and biochemical analysis of purified human immunodeficiency virus type 1 produced from infected monocyte-derived macrophages. J. Virol..

[29] Jalaguier P., Cantin R., Maaroufi H., Tremblay M.J. (2015). Selective Acquisition of Host-Derived ICAM-1 by HIV-1 Is a Matrix-Dependent Process. J. Virol..

[30] Bos L. (1999). Beijerinck’s work on tobacco mosaic virus: Historical context and legacy. Philos. Trans. R. Soc. B Biol. Sci..

[31] Roingeard P. (2008). Viral detection by electron microscopy: Past, present and future. Biol. Cell.

[32] Goldsmith C.S., Miller S.E. (2009). Modern Uses of Electron Microscopy for Detection of Viruses. Clin. Microbiol. Rev..

[33] Morales-Kastresana A., Telford B., Musich T.A., McKinnon K., Clayborne C., Braig Z., Rosner A., Demberg T., Watson D.C., Karpova T.S. (2017). Labeling extracellular vesicles for nanoscale flow cytometry. Sci. Rep..

[34] Liu J., Bartesaghi A., Borgnia M.J., Sapiro G., Subramaniam S. (2008). Molecular architecture of native HIV-1 gp120 trimers. Nature.

[35] Julien J.-P., Cupo A., Sok D., Stanfield R.L., Lyumkis D., Deller M.C., Klasse P.-J., Burton D.R., Sanders R.W., Moore J.P. (2013). Crystal structure of a soluble cleaved HIV-1 envelope trimer. Science.

[36] Shafique S. (2017). Envelope Protein Structure of Zika Virus—A Target For Vaccine Development And Therapeutics. Timely Top. Clin. Immunol..

[37] Ingale A.G. (2014). Epitopes Identification for Vaccine Design and Structural Aspects of Dengue Virus 3 Envelope Protein. Biochem. Physiol. Open Access.

[38] Pejchal R., Wilson I.A. (2010). Structure-based vaccine design in HIV: Blind men and the elephant?. Curr. Pharm. Des..

[39] Shirato K., Miyoshi H., Goto A., Ako Y., Ueki T., Kariwa H., Takashima I. (2004). Viral envelope protein glycosylation is a molecular determinant of the neuroinvasiveness of the New York strain of West Nile virus. J. Gen. Virol..

[40] Domon B., Aebersold R. (2006). Mass spectrometry and protein analysis. Science.

[41] Aebersold R., Mann M. (2003). Mass spectrometry-based proteomics. Nature.

[42] Link A.J., Eng J., Schieltz D.M., Carmack E., Mize G.J., Morris D.R., Garvik B.M., Yates J.R. (1999). Direct analysis of protein complexes using mass spectrometry. Nat. Biotechnol..

[43] Zheng J., Sugrue R.J., Tang K. (2011). Mass spectrometry based proteomic studies on viruses and hosts—A review. Anal. Chim. Acta.

[44] Ott D.E. (2002). Potential roles of cellular proteins in HIV-1. Rev. Med. Virol..

[45] Cross T.G., Hornshaw M.P. (2016). Can LC and LC-MS ever replace immunoassays?. J. Appl. Bioanal..

[46] Peeters B., Herijgers P., Beuselinck K., Peetermans W.E., Herregods M.-C., Desmet S., Lagrou K. (2016). Comparison of PCR-Electrospray Ionization Mass Spectrometry with 16S rRNA PCR and Amplicon Sequencing for Detection of Bacteria in Excised Heart Valves. J. Clin. Microbiol..

[47] Li Y., Holzgreve W., Kiefer V., Hahn S. (2006). MALDI-TOF Mass Spectrometry Compared with Real-Time PCR for Detection of Fetal Cell-Free DNA in Maternal Plasma. Clin. Chem..

[48] Loret S., Guay G., Lippe R. (2008). Comprehensive Characterization of Extracellular Herpes Simplex Virus Type 1 Virions. J. Virol..

[49] Shaw M.L., Stone K.L., Colangelo C.M., Gulcicek E.E., Palese P. (2008). Cellular Proteins in Influenza Virus Particles. PLoS Pathog..

[50] Saphire A.C.S., Gallay P.A., Bark S.J. (2006). Proteomic Analysis of Human Immunodeficiency Virus Using Liquid Chromatography/Tandem Mass Spectrometry Effectively Distinguishes Specific Incorporated Host Proteins. J. Proteome Res..

[51] Kannagi M., Kiyotaki M., King N.W., Lord C.I., Letvin N.L. (1987). Simian Immunodeficiency Virus Induces Expression of Class II Major Histocompatibility Complex Structures on Infected Target Cells In Vitro. J. Virol..

[52] Frank I., Stoiber H., Godar S., Stockinger H., Steindl F., Katinger H.W.D., Dierich M.P. (1996). Acquisition of host cell-surface-derived molecules by HIV-1. AIDS.

[53] Montefiori D.C., Cornell R.J., Zhou J.Y., Zhou J.T., Hirsch V.M., Johnson P.R. (1994). Complement control proteins, CD46, CD55, and CD59, as common surface constituents of human and simian immunodeficiency viruses and possible targets for vaccine protection. Virology.

[54] Cantin R., Fortin J.-F., Tremblay M. (1996). The Amount of Host HLA-DR Proteins Acquired by HIV-1 Is Virus Strain- and Cell Type-Specific. Virology.

[55] Capobianchi M.R., Fais S., Castilletti C., Gentile M., Ameglio F., Dianzani F. (1994). A Simple and Reliable Method to Detect Cell Membrane Proteins on Infectious Human Immunodeficiency Virus Type 1 Particles. J. Infect. Dis..

[56] Lawn S.D., Roberts B.D., Griffin G.E., Folks T.M., Butera S.T. (2000). Cellular Compartments of Human Immunodeficiency Virus Type 1 Replication In Vivo: Determination by Presence of Virion-Associated Host Proteins and Impact of Opportunistic Infection. J. Virol..

[57] Bounou S., Giguère J.-F., Cantin R., Gilbert C., Imbeault M., Martin G., Tremblay M.J. (2004). The importance of virus-associated host ICAM-1 in human immunodeficiency virus type 1 dissemination depends on the cellular context. FASEB J..

[58] Esser M.T., Graham D.R., Coren L.V., Trubey C.M., Bess J.W., Arthur L.O., Ott D.E., Lifson J.D. (2001). Differential Incorporation of CD45, CD80 (B7-1), CD86 (B7-2), and Major Histocompatibility Complex Class I and II Molecules into Human Immunodeficiency Virus Type 1 Virions and Microvesicles: Implications for Viral Pathogenesis and Immune Regulation. J. Virol..

[59] Linde M.E., Colquhoun D.R., Mohien C.U., Kole T., Aquino V., Cotter R., Edwards N., Hildreth J.E.K., Graham D.R. (2013). The Conserved Set of Host Proteins Incorporated into HIV-1 Virions Suggests a Common Egress Pathway in Multiple Cell Types. J. Proteome Res..

[60] Bess J.W., Gorelick R.J., Bosche W.J., Henderson L.E., Arthur L.O. (1997). Microvesicles are a source of contaminating cellular proteins found in purified HIV-1 preparations. Virology.

[61] Martin G., Tremblay M.J. (2004). HLA-DR, ICAM-1, CD40, CD40L, and CD86 are incorporated to a similar degree into clinical human immunodeficiency virus type 1 variants expanded in natural reservoirs such as peripheral blood mononuclear cells and human lymphoid tissue cultured ex vivo. Clin. Immunol..

[62] Frank I., Kacani L., Stoiber H., Steindl F., Romani N., Dierich M.P. (1999). Human Immunodeficiency Virus Type 1 Derived from Cocultures of Immature Dendritic Cells with Autologous T Cells Carries T-Cell-Specific Molecules on Its Surface and Is Highly Infectious. J. Virol..

[63] Thibault S., Tardif M.R., Gilbert C., Tremblay M.J. (2007). Virus-associated host CD62L increases attachment of human immunodeficiency virus type 1 to endothelial cells and enhances trans infection of CD4+ T lymphocytes. J. Gen. Virol..

[64] Bastiani L., Laal S., Kim M., Zolla-Pazner S. (1997). Host Cell-Dependent Alterations in Envelope Components of Human Immunodeficiency Virus Type 1 Virions. J. Virol..

[65] Macey M.G., Macey M.G. (2007). Flow Cytometry.

[66] Jaroszeski M.J., Radcliff G. (1999). Fundamentals of flow cytometry. Mol. Biotechnol..

[67] Betters D.M. (2015). Use of flow cytometry in clinical practice. J. Adv. Pract. Oncol..

[68] Brown M., Wittwer C. (2000). Flow cytometry: Principles and clinical applications in hematology. Clin. Chem..

[69] Steen H.B. (2004). Flow cytometer for measurement of the light scattering of viral and other submicroscopic particles. Cytom. Part J. Int. Soc. Anal. Cytol..

[70] Bonar M.M., Tilton J.C. (2017). High sensitivity detection and sorting of infectious human immunodeficiency virus (HIV-1) particles by flow virometry. Virology.

[71] Hercher M., Mueller W., Shapiro H.M. (1979). Detection and discrimination of individual viruses by flow cytometry. J. Histochem. Cytochem..

[72] Marie D., Brussaard C.P.D., Thyrhaug R., Bratbak G., Vaulot D. (1999). Enumeration of Marine Viruses in Culture and Natural Samples by Flow Cytometry. Appl. Environ. Microbiol..

[73] Brussaard C.P.D., Marie D., Bratbak G. (2000). Flow cytometric detection of viruses. J. Virol. Methods.

[74] Brussaard C.P.D. (2004). Optimization of Procedures for Counting Viruses by Flow Cytometry. Appl. Environ. Microbiol..

[75] Arakelyan A., Fitzgerald W., Margolis L., Grivel J.-C. (2013). Nanoparticle-based flow virometry for the analysis of individual virions. J. Clin. Investig..

[76] Tang V.A., Renner T.M., Fritzsche A.K., Burger D., Langlois M.-A. (2017). Single-Particle Discrimination of Retroviruses from Extracellular Vesicles by Nanoscale Flow Cytometry. Sci. Rep..

[77] Tang V.A., Renner T.M., Varette O., Le Boeuf F., Wang J., Diallo J.-S., Bell J.C., Langlois M.-A. (2016). Single-particle characterization of oncolytic vaccinia virus by flow virometry. Vaccine.

[78] Reyes J.L.Z., Aguilar H.C. (2018). Flow virometry as a tool to study viruses. Methods.

[79] Lippé R. (2018). Flow virometry: A powerful tool to functionally characterize viruses. J. Virol..

[80] Nolte E., Cremer T., Gallo R.C., Margolis L.B. (2016). Extracellular vesicles and viruses: Are they close relatives?. Proc. Natl. Acad. Sci. USA.

[81] Musich T., Jones J.C., Keele B.F., Jenkins L.M.M., Demberg T., Uldrick T.S., Yarchoan R., Robert-Guroff M. (2017). Flow virometric sorting and analysis of HIV quasispecies from plasma. JCI Insight.

[82] Gluschankof P., Mondor I., Gelderblom H.R., Sattentau Q.J. (1997). Cell membrane vesicles are a major contaminant of gradient-enriched human immunodeficiency virus type-1 preparations. Virology.

[83] Tang V.A., Fritzsche A.K., Renner T.M., Burger D., Brittain G.C., Lannigan J.A., Ouellet C., van der Pol E., Langlois M.-A. Retroviruses as Fluorescent Reference Particles for Nanoscale Flow Cytometry. BioRxiv.

[84] Van der Pol E., Coumans F.A.W., Sturk A., Nieuwland R., van Leeuwen T.G. (2014). Refractive Index Determination of Nanoparticles in Suspension Using Nanoparticle Tracking Analysis. Nano Lett..

[85] Chandler W.L., Yeung W., Tait J.F. (2011). A new microparticle size calibration standard for use in measuring smaller microparticles using a new flow cytometer. J. Thromb. Haemost..

[86] FCM PASS—Joshua A. Welsh. http://www.joshuawelsh.co.uk/scatter-diameter-software/.

[87] Rosetta Calibration. https://www.exometry.com/products/rosetta-calibration.

[88] Boily M.-C., Baggaley R.F., Wang L., Masse B., White R.G., Hayes R.J., Alary M. (2009). Heterosexual risk of HIV-1 infection per sexual act: Systematic review and meta-analysis of observational studies. Lancet Infect. Dis..

[89] Saarloos M.-N., Sullivan B.L., Czerniewski M.A., Parameswar K.D., Spear G.T. (1997). Detection of HLA-DR Associated with Monocytotropic, Primary, and Plasma Isolates of Human Immunodeficiency Virus Type. J. Virol..

[90] Cantin R., Martin G., Tremblay M.J. (2001). A novel virus capture assay reveals a differential acquisition of host HLA-DR by clinical isolates of human immunodeficiency virus type 1 expanded in primary human cells depending on the nature of producing cells and the donor source. J. Gen. Virol..

[91] Scheppler J.A., Nicholson J.K., Swan D.C., Ahmed-Ansari A., McDougal J.S. (1989). Down-modulation of MHC-I in a CD4+ T cell line, CEM-E5, after HIV-1 infection. J. Immunol..

[92] Kerkau T., Bacik I., Bennink J.R., Yewdell J.W., Hünig T., Schimpl A., Schubert U. (1997). The Human Immunodeficiency Virus Type 1 (HIV-1) Vpu Protein Interferes with an Early Step in the Biosynthesis of Major Histocompatibility Complex (MHC) Class I Molecules. J. Exp. Med..

[93] Cohen G.B., Gandhi R.T., Davis D.M., Mandelboim O., Chen B.K., Strominger J.L., Baltimore D. (1999). The Selective Downregulation of Class I Major Histocompatibility Complex Proteins by HIV-1 Protects HIV-Infected Cells from NK Cells. Immunity.

[94] Bonaparte M.I. (2004). Killing of human immunodeficiency virus-infected primary T-cell blasts by autologous natural killer cells is dependent on the ability of the virus to alter the expression of major histocompatibility complex class I molecules. Blood.

[95] Bengtsson A., Joergensen L., Barbati Z.R., Craig A., Hviid L., Jensen A.T.R. (2013). Transfected HEK293 Cells Expressing Functional Recombinant Intercellular Adhesion Molecule 1 (ICAM-1)—A Receptor Associated with Severe Plasmodium falciparum Malaria. PLoS ONE.

[96] Staunton D.E., Merluzzi V.J., Rothlein R., Barton R., Marlin S.D., Springer T.A. (1989). A cell adhesion molecule, ICAM-1, is the major surface receptor for rhinoviruses. Cell.

[97] Marlin S.D., Springer T.A. (1987). Purified intercellular adhesion molecule-1 (ICAM-1) is a ligand for lymphocyte function-associated antigen 1 (LFA-1). Cell.

[98] Lifson J.D., Reyes G.R., McGrath M.S., Stein B.S., Engleman E.G. (1986). AIDS retrovirus induced cytopathology: Giant cell formation and involvement of CD4 antigen. Science.

[99] Hildreth J.E.K., Orentas R.J. (1989). Involvement of a Leukocyte Adhesion Receptor (LFA-1) in HIV-Induced Syncytium Formation. Science.

[100] Butini L., De Fougerolles A.R., Vaccarezza M., Graziosi C., Cohen D.I., Montroni M., Springer T.A., Pantaleo G., Fauci A.S. (1994). Intercellular adhesion molecules (ICAM)-1 ICAM-2 and ICAM-3 function as counter-receptors for lymphocyte function-associated molecule 1 in human immunodeficiency virus-mediated syncytia formation. Eur. J. Immunol..

[101] Fecondo J.V., Pavuk N.C., Silburn K.A., Read D.M.Y., Mansell A.S., Boyd A.W., McPHEE D.A. (1993). Synthetic Peptide Analogs of Intercellular Adhesion Molecule 1 (ICAM-1) Inhibit HIV-1 Replication in MT-2 Cells. AIDS Res. Hum. Retrovir..

[102] Sommerfelt M.A., Asjo B. (1995). Intercellular adhesion molecule 3, a candidate human immunodeficiency virus type 1 co-receptor on lymphoid and monocytoid cells. J. Gen. Virol..

[103] Briskin M., Winsor-Hines D., Shyjan A., Cochran N., Bloom S., Wilson J., McEvoy L.M., Butcher E.C., Kassam N., Mackay C.R. (1997). Human mucosal addressin cell adhesion molecule-1 is preferentially expressed in intestinal tract and associated lymphoid tissue. Am. J. Pathol..

[104] Arthos J., Cicala C., Martinelli E., Macleod K., Van Ryk D., Wei D., Xiao Z., Veenstra T.D., Conrad T.P., Lempicki R.A. (2008). HIV-1 envelope protein binds to and signals through integrin α4β7, the gut mucosal homing receptor for peripheral T cells. Nat. Immunol..

[105] Wang X., Xu H., Gill A.F., Pahar B., Kempf D., Rasmussen T., Lackner A.A., Veazey R.S. (2009). Monitoring α4β7 integrin expression on circulating CD4+ T cells as a surrogate marker for tracking intestinal CD4+ T-cell loss in SIV infection. Mucosal Immunol..

[106] Sivro A., Schuetz A., Sheward D., Joag V., Yegorov S., Liebenberg L.J., Yende-Zuma N., Stalker A., Mwatelah R.S., Selhorst P. (2018). Integrin α4β7 expression on peripheral blood CD4+ T cells predicts HIV acquisition and disease progression outcomes. Sci. Transl. Med..

[107] Brenchley J.M., Schacker T.W., Ruff L.E., Price D.A., Taylor J.H., Beilman G.J., Nguyen P.L., Khoruts A., Larson M., Haase A.T. (2004). CD4+ T Cell Depletion during all Stages of HIV Disease Occurs Predominantly in the Gastrointestinal Tract. J. Exp. Med..

[108] Cicala C., Martinelli E., McNally J.P., Goode D.J., Gopaul R., Hiatt J., Jelicic K., Kottilil S., Macleod K., O’Shea A. (2009). The integrin α4β7 forms a complex with cell-surface CD4 and defines a T-cell subset that is highly susceptible to infection by HIV-1. Proc. Natl. Acad. Sci. USA.

[109] McKinnon L.R., Kaul R. (2012). Quality and quantity: Mucosal CD4+ T cells and HIV susceptibility. Curr. Opin. HIV AIDS.

[110] Charles A Janeway J., Travers P., Walport M., Shlomchik M.J. (2001). The complement system and innate immunity. Immunobiology: The Immune System in Health and Disease.

[111] Walport M.J. (2001). Complement. N. Engl. J. Med..

[112] Weiss L., Okada N., Haeffner-Cavaillon N., Hattori T., Faucher C., Kazatchkine M.D., Okada H. (1992). Decreased Expression of the Membrane Inhibitor of Complement-Mediated Cytolysis CD59 on T-lymphocytes of HIV-Infected Patients. AIDS.

[113] Terpos E., Sarantopoulos A., Kouramba A., Katsarou O., Stavropoulos J., Masouridi S., Karafoulidou A., Meletis J. (2008). Reduction of CD55 and/or CD59 in red blood cells of patients with HIV infection. Med. Sci. Monit..

[114] Banapour B., Sernatinger J., Levy J.A. (1986). The AIDS-associated retrovirus is not sensitive to lysis or inactivation by human serum. Virology.

[115] Saifuddin M. (1995). Role of virion-associated glycosylphosphatidylinositol-linked proteins CD55 and CD59 in complement resistance of cell line-derived and primary isolates of HIV-1. J. Exp. Med..

[116] Foy T.M., Aruffo A., Bajorath J., Buhlmann J.E., Noelle R.J. (1996). Immune Regulation by Cd40 and Its Ligand Gp39. Annu. Rev. Immunol..

[117] Grewal I.S., Flavell R.A. (1996). A central role of CD40 ligand in the regulation of CD4+ T-cell responses. Immunol. Today.

[118] Maurais É., Cantin R., Tremblay M.J. (2009). Human immunodeficiency virus type 1-anchored CD40 ligand induces secretion of the chemokine interleukin-8 by human primary macrophages. Virology.

[119] Martin G., Roy J., Barat C., Ouellet M., Gilbert C., Tremblay M.J. (2007). Human Immunodeficiency Virus Type 1-Associated CD40 Ligand Transactivates B Lymphocytes and Promotes Infection of CD4+ T Cells. J. Virol..

[120] Nguyen D.G., Booth A., Gould S.J., Hildreth J.E.K. (2003). Evidence That HIV Budding in Primary Macrophages Occurs through the Exosome Release Pathway. J. Biol. Chem..

[121] Roberts B.D., Butera S.T. (1999). Host protein incorporation is conserved among diverse HIV-1 subtypes. AIDS.

[122] Gelderblom H.R. (1997). Fine Structure of HIV and SIV.

[123] Nguyen D.H., Hildreth J.E.K. (2000). Evidence for Budding of Human Immunodeficiency Virus Type 1 Selectively from Glycolipid-Enriched Membrane Lipid Rafts. J. Virol..

[124] Ono A., Freed E.O. (2001). Plasma membrane rafts play a critical role in HIV-1 assembly and release. Proc. Natl. Acad. Sci. USA.

[125] Brown D.A., London E. (1998). Functions of Lipid Rafts in Biological Membranes. Annu. Rev. Cell Dev. Biol..

[126] Simons K., Ikonen E. (1997). Functional rafts in cell membranes. Nature.

[127] Liao Z., Cimakasky L.M., Hampton R., Nguyen D.H., Hildreth J.E.K. (2001). Lipid Rafts and HIV Pathogenesis: Host Membrane Cholesterol Is Required for Infection by HIV Type 1. AIDS Res. Hum. Retrovir..

[128] Chazal N., Gerlier D. (2003). Virus Entry, Assembly, Budding, and Membrane Rafts. Microbiol. Mol. Biol. Rev..

[129] Aloia R.C., Tian H., Jensen F.C. (1993). Lipid composition and fluidity of the human immunodeficiency virus envelope and host cell plasma membranes. Proc. Natl. Acad. Sci. USA.

[130] Guyader M., Kiyokawa E., Abrami L., Turelli P., Trono D. (2002). Role for Human Immunodeficiency Virus Type 1 Membrane Cholesterol in Viral Internalization. J. Virol..

[131] Liao Z., Graham D.R., Hildreth J.E.K. (2003). Lipid Rafts and HIV Pathogenesis: Virion-Associated Cholesterol Is Required for Fusion and Infection of Susceptible Cells. AIDS Res. Hum. Retrovir..

[132] Graham D.R.M., Chertova E., Hilburn J.M., Arthur L.O., Hildreth J.E.K. (2003). Cholesterol Depletion of Human Immunodeficiency Virus Type 1 and Simian Immunodeficiency Virus with -Cyclodextrin Inactivates and Permeabilizes the Virions: Evidence for Virion-Associated Lipid Rafts. J. Virol..

[133] Popik W., Alce T.M., Au W.-C. (2002). Human Immunodeficiency Virus Type 1 Uses Lipid Raft-Colocalized CD4 and Chemokine Receptors for Productive Entry into CD4+ T Cells. J. Virol..

[134] Aloia R.C., Jensen F.C., Curtain C.C., Mobley P.W., Gordon L.M. (1988). Lipid composition and fluidity of the human immunodeficiency virus. Proc. Natl. Acad. Sci. USA.

[135] Jury E.C., Flores-Borja F., Kabouridis P.S. (2007). Lipid rafts in T cell signalling and disease. Semin. Cell Dev. Biol..

[136] Zhang M., Moran M., Round J., Low T.A., Patel V.P., Tomassian T., Hernandez J.D., Miceli M.C. (2005). CD45 Signals outside of Lipid Rafts to Promote ERK Activation, Synaptic Raft Clustering, and IL-2 Production. J. Immunol..

[137] Nakano A., Harada T., Morikawa S., Kato Y. (1990). Expression of leukocyte common antigen (CD45) on various human leukemia/lymphoma cell lines. Pathol. Int..

[138] Ono A., Freed E.O. (2005). Role of Lipid Rafts in Virus Replication**This chapter was written by Akira Ono and Eric O. Freed in their personal capacity. The views expressed in this chapter do not necessarily reflect the views of the NIH, DHHS, or the United States. Advances in Virus Research.

[139] Janes P.W., Ley S.C., Magee A.I. (1999). Aggregation of Lipid Rafts Accompanies Signaling via the T Cell Antigen Receptor. J. Cell Biol..

[140] Parolini I., Topa S., Sorice M., Pace A., Ceddia P., Montesoro E., Pavan A., Lisanti M.P., Peschle C., Sargiacomo M. (1999). Phorbol Ester-induced Disruption of the CD4-Lck Complex Occurs within a Detergent-resistant Microdomain of the Plasma Membrane: Involvement of the Translocation of Activated Protein Kinase C Isoforms. J. Biol. Chem..

[141] Cinek T., Horejsi V. (1992). The nature of large noncovalent complexes containing glycosyl-phosphatidylinositol-anchored membrane glycoproteins and protein tyrosine kinases. J. Immunol..

[142] Millán J., Cerny J., Horejsi V., Alonso M.A. (1999). CD4 segregates into specific detergent-resistant T-cell membrane microdomains. Tissue Antigens.

[143] Aiken C. (1994). Nef induces CD4 endocytosis: Requirement for a critical dileucine motif in the membrane-proximal CD4 cytoplasmic domain. Cell.

[144] Willey R.L., Maldarelli F., Martin M.A., Strebel K. (1992). Human immunodeficiency virus type 1 Vpu protein induces rapid degradation of CD4. J. Virol..

[145] Lu Y.E., Kielian M. (2000). Semliki Forest Virus Budding: Assay, Mechanisms, and Cholesterol Requirement. J. Virol..

[146] Scheiffele P., Rietveld A., Wilk T., Simons K. (1999). Influenza Viruses Select Ordered Lipid Domains during Budding from the Plasma Membrane. J. Biol. Chem..

[147] Orenstein J.M., Meltzer M.S., Phipps T., Gendelman H.E. (1988). Cytoplasmic assembly and accumulation of human immunodeficiency virus types 1 and 2 in recombinant human colony-stimulating factor-1-treated human monocytes: An ultrastructural study. J. Virol..

[148] Venzke S., Keppler O.T. (2006). Role of macrophages in HIV infection and persistence. Expert Rev. Clin. Immunol..

[149] Cantin R., Methot S., Tremblay M.J. (2005). Plunder and Stowaways: Incorporation of Cellular Proteins by Enveloped Viruses. J. Virol..

[150] Gould S.J., Booth A.M., Hildreth J.E. (2003). The Trojan exosome hypothesis. Proc. Natl. Acad. Sci. USA.

[151] Wubbolts R., Leckie R.S., Veenhuizen P.T.M., Schwarzmann G., Möbius W., Hoernschemeyer J., Slot J.-W., Geuze H.J., Stoorvogel W. (2003). Proteomic and Biochemical Analyses of Human B Cell-derived Exosomes: Potential Implications for Their Function and Multivesicular Body Formation. J. Biol. Chem..

[152] Raposo G., Moore M., Innes D., Leijendekker R., Leigh-Brown A., Benaroch P., Geuze H. (2002). Human macrophages accumulate HIV-1 particles in MHC II compartments. Traffic.

[153] Pelchen-Matthews A., Kramer B., Marsh M. (2003). Infectious HIV-1 assembles in late endosomes in primary macrophages. J. Cell Biol..

[154] Pelchen-Matthews A., Raposo G., Marsh M. (2004). Endosomes, exosomes and Trojan viruses. Trends Microbiol..

[155] Cassol E., Alfano M., Biswas P., Poli G. (2006). Monocyte-derived macrophages and myeloid cell lines as targets of HIV-1 replication and persistence. J. Leukoc. Biol..

[156] Stolp B., Fackler O.T. (2011). How HIV Takes Advantage of the Cytoskeleton in Entry and Replication. Viruses.

[157] Iyengar S., Hildreth J.E.K., Schwartz D.H. (1998). Actin-Dependent Receptor Colocalization Required for Human Immunodeficiency Virus Entry into Host Cells. J. Virol..

[158] Gladnikoff M., Shimoni E., Gov N.S., Rousso I. (2009). Retroviral Assembly and Budding Occur through an Actin-Driven Mechanism. Biophys. J..

[159] Jiménez-Baranda S., Gómez-Moutón C., Rojas A., Martínez-Prats L., Mira E., Lacalle R.A., Valencia A., Dimitrov D.S., Viola A., Delgado R. (2007). Filamin-A regulates actin-dependent clustering of HIV receptors. Nat. Cell Biol..

[160] Audoly G., Popoff M.R., Gluschankof P. (2005). Involvement of a small GTP binding protein in HIV-1 release. Retrovirology.

[161] Sasaki H., Nakamura M., Ohno T., Matsuda Y., Yuda Y., Nonomura Y. (1995). Myosin-actin interaction plays an important role in human immunodeficiency virus type 1 release from host cells. Proc. Natl. Acad. Sci. USA.

[162] Liu B., Dai R., Tian C.-J., Dawson L., Gorelick R., Yu X.-F. (1999). Interaction of the human immunodeficiency virus type 1 nucleocapsid with actin. J. Virol..

[163] Rey O., Canon J., Krogstad P. (1996). HIV-1 Gag protein associates with F-actin present in microfilaments. Virology.

[164] Martinez N.W., Xue X., Berro R.G., Kreitzer G., Resh M.D. (2008). Kinesin KIF4 Regulates Intracellular Trafficking and Stability of the Human Immunodeficiency Virus Type 1 Gag Polyprotein. J. Virol..

[165] Ospina Stella A., Turville S. (2018). All-round manipulation of the actin cytoskeleton by HIV. Viruses.

[166] Dai L., Lidie K.B., Chen Q., Adelsberger J.W., Zheng X., Huang D., Yang J., Lempicki R.A., Rehman T., Dewar R.L. (2013). IL-27 inhibits HIV-1 infection in human macrophages by down-regulating host factor SPTBN1 during monocyte to macrophage differentiation. J. Exp. Med..

[167] Swaminathan S., Dai L., Lane H.C., Imamichi T. (2013). Evaluating the potential of IL-27 as a novel therapeutic agent in HIV-1 infection. Cytokine Growth Factor Rev..

[168] Del Río-Iñiguez I., Vázquez-Chávez E., Cuche C., Di Bartolo V., Bouchet J., Alcover A. (2018). HIV-1 Nef Hijacks Lck and Rac1 Endosomal Traffic to Dually Modulate Signaling-Mediated and Actin Cytoskeleton–Mediated T Cell Functions. J. Immunol..

[169] Vérollet C., Le Cabec V., Maridonneau-Parini I. (2015). HIV-1 infection of T lymphocytes and macrophages affects their migration via Nef. Front. Immunol..

[170] Martin G., Beauséjour Y., Thibodeau J., Tremblay M.J. (2005). Envelope glycoproteins are dispensable for insertion of host HLA-DR molecules within nascent human immunodeficiency virus type 1 particles. Virology.

[171] Beausejour Y., Tremblay M.J. (2004). Interaction between the Cytoplasmic Domain of ICAM-1 and Pr55Gag Leads to Acquisition of Host ICAM-1 by Human Immunodeficiency Virus Type 1. J. Virol..

[172] Poon D.T.K., Coren L.V., Ott D.E. (2000). Efficient Incorporation of HLA Class II onto Human Immunodeficiency Virus Type 1 Requires Envelope Glycoprotein Packaging. J. Virol..

[173] Fortin J.-F.O., Cantin R.J., Tremblay M.J. (1998). T Cells Expressing Activated LFA-1 Are More Susceptible to Infection with Human Immunodeficiency Virus Type 1 Particles Bearing Host-Encoded ICAM-. J. Virol..

[174] Fortin J.-F., Cantin R., Bergeron M.G., Tremblay M.J. (2000). Interaction between Virion-Bound Host Intercellular Adhesion Molecule-1 and the High-Affinity State of Lymphocyte Function-Associated Antigen-1 on Target Cells Renders R5 and X4 Isolates of Human Immunodeficiency Virus Type 1 More Refractory to Neutralization. Virology.

[175] Cosma A., Blanc D., Braun J., Quillent C., Barassi C., Moog C., Klasen S., Spire B., Scarlatti G., Pesenti E. (1999). Enhanced HIV infectivity and changes in GP120 conformation associated with viral incorporation of human leucocyte antigen class I molecules. AIDS.

[176] Bounou S., Leclerc J.E., Tremblay M.J. (2002). Presence of Host ICAM-1 in Laboratory and Clinical Strains of Human Immunodeficiency Virus Type 1 Increases Virus Infectivity and CD4+-T-Cell Depletion in Human Lymphoid Tissue, a Major Site of Replication In Vivo. J. Virol..

[177] Fujiwara M., Tsunoda R., Shigeta S., Yokota T., Baba M. (1999). Human Follicular Dendritic Cells Remain Uninfected and Capture Human Immunodeficiency Virus Type 1 through CD54-CD11a Interaction. J. Virol..

[178] Heath S.L., Tew J.G., Tew J.G., Szakal A.K., Burton G.F. (1995). Follicular dendritic cells and human immunodeficiency virus infectivity. Nature.

[179] Hioe C.E., Chien P.C., Lu C., Springer T.A., Wang X.-H., Bandres J., Tuen M. (2001). LFA-1 Expression on Target Cells Promotes Human Immunodeficiency Virus Type 1 Infection and Transmission. J. Virol..

[180] Cantin R., Fortin J.-F., Lamontagne G., Tremblay M. (1997). The presence of host-derived HLA-DR1 on human immunodeficiency virus type 1 increases viral infectivity. J. Virol..

[181] Cantin R., Fortin J.-F., Lamontagne G., Tremblay M. (1997). The acquisition of host-derived major histocompatibility complex class II glycoproteins by human immunodeficiency virus type 1 accelerates the process of virus entry and infection in human T-lymphoid cells. Blood.

[182] Rossio J.L., Bess J., Henderson L.E., Cresswell P., Arthur L.O. (1995). HLA class II on HIV particles is functional in superantigen presentation to human T cells: Implications for HIV pathogenesis. AIDS Res. Hum. Retrovir..

[183] Euler Z., Alter G. (2014). Exploring the Potential of Monoclonal Antibody Therapeutics for HIV-1 Eradication. AIDS Res. Hum. Retrovir..

[184] Li C., Jin W., Du T., Wu B., Liu Y., Shattock R.J., Hu Q. (2014). Binding of HIV-1 virions to α4β7 expressing cells and impact of antagonizing α4β7 on HIV-1 infection of primary CD4+ T cells. Virol. Sin..

[185] Perez L.G., Chen H., Liao H.-X., Montefiori D.C. (2014). Envelope Glycoprotein Binding to the Integrin 4 7 Is Not a General Property of Most HIV-1 Strains. J. Virol..

[186] Santangelo P.J., Cicala C., Byrareddy S.N., Ortiz K.T., Little D., Lindsay K.E., Gumber S., Hong J.J., Jelicic K., Rogers K.A. (2018). Early treatment of SIV+ macaques with an α4 β7 mAb alters virus distribution and preserves CD4+ T cells in later stages of infection. Mucosal Immunol..

[187] Uzzan M., Tokuyama M., Rosenstein A.K., Tomescu C., SahBandar I.N., Ko H.M., Leyre L., Chokola A., Kaplan-Lewis E., Rodriguez G. (2018). Anti-α4β7 therapy targets lymphoid aggregates in the gastrointestinal tract of HIV-1 infected individuals. Sci. Transl. Med..

[188] Fauci A.S. (2017). An HIV Vaccine Is Essential for Ending the HIV/AIDS Pandemic. JAMA.

[189] Das K., Arnold E. (2013). HIV-1 Reverse Transcriptase and Antiviral Drug Resistance (Part 1 of 2). Curr. Opin. Virol..

[190] Lehner T., Shearer G.M., Hackett C.J., Schultz A., Sharma O.K. (2000). Alloimmunization as a Strategy for Vaccine Design against HIV/AIDS. AIDS Res. Hum. Retrovir..

[191] Shearer G.M., Clerici M., Dalgleish A. (1993). Alloimmunization as an AIDS Vaccine?. Science.

[192] Wang Y. (2018). Development of a human leukocyte antigen-based HIV vaccine. F1000Research.

[193] Mörner A., Jansson M., Bunnik E.M., Schøller J., Vaughan R., Wang Y., Montefiori D.C., Otting N., Bontrop R., Bergmeier L.A. (2011). Immunization with Recombinant HLA Classes I and II, HIV-1 gp140, and SIV p27 Elicits Protection against Heterologous SHIV Infection in Rhesus Macaques. J. Virol..

[194] Yang G.-B., Wang Y., Babaahmady K., Schøller J., Rahman D., Bunnik E., Spallek R., Zong C.-M., Duan J.-Z., Qin C. (2012). Immunization with recombinant macaque major histocompatibility complex class I and II and human immunodeficiency virus gp140 inhibits simian–human immunodeficiency virus infection in macaques. J. Gen. Virol..

[195] Wang Y., Tao L., Mitchell E., Bravery C., Berlingieri P., Armstrong P., Vaughan R., Underwood J., Lehner T. (1999). Allo-immunization elicits CD8+ T cell-derived chemokines, HIV suppressor factors and resistance to HIV infection in women. Nat. Med..

[196] Jeffrey P.-L., Wang Y., Seidl T., Babaahmady K., Vaughan R., Lehner T. (2009). The effect of allogeneic in vitro stimulation and in vivo immunization on memory CD4^+^ T-cell APOBEC3G expression and HIV-1 infectivity: Clinical immunology. Eur. J. Immunol..

[197] Peters B., Whittall T., Babaahmady K., Gray K., Vaughan R., Lehner T. (2004). Effect of heterosexual intercourse on mucosal alloimmunisation and resistance to HIV-1 infection. Lancet.

[198] Zamani C., Elzey J.D., Hildreth J.E. (2013). Greater ethnic diversity correlates with lower HIV prevalence in Africa: Justification for an alloimmunity vaccine. HIVAIDS.

[199] Murphey-Corb M., Martin L.N., Davison-Fairburn B., Montelaro R.C., Miller M., West M., Ohkawa S., Baskin G.B., Zhang J.Y., Putney S.D. (1989). A formalin-inactivated whole SIV vaccine confers protection in macaques. Science.

[200] Carlson J.R., McGraw T.P., Keddie E., Yee J.L., Rosenthal A., Langlois A.J., Dickover R., Donovan R., Luciw P.A., Jennings M.B. (1990). Vaccine Protection of Rhesus Macaques Against Simian Immunodeficiency Virus Infection. AIDS Res. Hum. Retrovir..

[201] Dormont D., Le Grand R., Cranage M., Greenaway P., Hunsmann G., Stahl-Hennig C., Rossi G., Verani P., Stott J., The European Concerted Action on ‘Macaque Models for AIDS Research’ (1995). Protection of macaques against simian immunodeficiency virus infection with inactivated vaccines: Comparison of adjuvants, doses and challenge viruses. Vaccine.

[202] Stott E.J., Taffs F., Kitchin P., Chan W.L., Mills K., Page M., Cranage M., Greenaway P. (1990). Preliminary report: Protection of cynomolgus macaques against simian immunodeficiency virus by fixed infected-cell vaccine. Lancet.

[203] Tardif M.R., Tremblay M.J. (2005). LFA-1 Is a Key Determinant for Preferential Infection of Memory CD4+ T Cells by Human Immunodeficiency Virus Type 1. J. Virol..

[204] Cheng L., Wang Q., Li G., Banga R., Ma J., Yu H., Yasui F., Zhang Z., Pantaleo G., Perreau M. (2018). TLR3 agonist and CD40-targeting vaccination induces immune responses and reduces HIV-1 reservoirs. J. Clin. Investig..

